# CircRNA circ_0015278 induces ferroptosis in lung adenocarcinoma through the miR-1228/P53 axis

**DOI:** 10.32604/or.2024.050835

**Published:** 2025-01-16

**Authors:** LIANGJIANG XIA, GUANGBIN LI, QINGWU ZHOU, YU FENG, HAITAO MA

**Affiliations:** 1 Department of Thoracic Surgery, The Fourth Affiliated Hospital of Soochow University, Suzhou, 215006, China; 2 Department of Thoracic Surgery, The First Affiliated Hospital of Soochow University, Suzhou, 215006, China; 3 The First Clinical Medical College of Nanchang University, The First Affiliated Hospital of Nanchang University, Nanchang, 330006, China

**Keywords:** Lung adenocarcinoma (LUAD), Circ_0015278, Ferroptosis, miR-1228, P53, SLC7A11

## Abstract

**Background:**

Circular RNAs play an important role in regulating lung adenocarcinoma (LUAD). Bioinformatics analysis identified circ_0015278 as differentially expressed in LUAD. However, the biological mechanism of circ_0015278 in LUAD has not been fully clarified, especially in ferroptosis.

**Materials and Methods:**

Bioinformatics analysis was employed to explore the downstream mechanisms of Circ_0015278, subsequently confirmed by luciferase reporter assays. The impact of Circ_0015278 on cell proliferation, migration, invasion, and ferroptosis was investigated through a loss-of-function experiment. A xenotransplantation mouse model elucidated the effect of Circ_0015278 on tumour growth.

**Results:**

Circ_0015278 exhibited downregulation in LUAD. It inhibited cell proliferation, migration, and invasion while promoting ferroptosis by interacting with miR-1228 to regulate P53 expression through a competitive endogenous RNA mechanism. Moreover, circ_0015278 suppressed tumour growth in mice.

**Conclusions:**

Circ_0015278 was identified as a novel factor promoting ferroptosis in LUAD. Furthermore, it suppressed the malignant progression of LUAD through the miR-1228/P53 axis.

## Introduction

Lung adenocarcinoma (LUAD) stands out as a prevalent histological subtype and the primary contributor to mortality in non-small cell lung cancer (NSCLC), a prevalent form of lung malignancy [1,2]. Despite advancements in therapeutic modalities such as surgery, radiation therapy, chemotherapy, and immunotherapy, LUAD patients consistently exhibit poor survival outcomes. The progression, metastasis, and development of resistance to pharmacological agents by the tumor are critical factors influencing the prognosis of LUAD patients. Recent studies have revealed that the induction of ferroptosis in malignant cells impedes cancer growth and metastasis, as well as enhances the sensitivity of anti-cancer drugs [3,4].

Ferroptosis, an innovative form of programmed cell death, is iron-dependent. And distinguishes itself from apoptosis, necrosis, and autophagy [5,6]. The System Xc-composed of two subunits, solute carrier family 7 membrane 11 (SLC7A11) and solute carrier family 3 member 2 (SLC3A2), is an essential antioxidant system. Decrease of its activity leads to the accruement of lipid reactive oxygen species (ROS) by inhibiting the activity of glutathione peroxidase 4 (GPX4), which further causes oxidative damage and ferroptosis. By taking up cystine and promoting glutathione biosynthesis, SLC7A11 effectively inhibits ferroptosis [7]. Additionally, P53 has been found to suppress SLC7A11 expression and induce ferroptosis to overcome drug resistance [8].

Emerging evidence indicates that non-coding RNAs act as competing endogenous RNAs (ceRNA) by competing with various miRNAs [9,10]. CircRNAs abundant in miRNA binding sites (MREs), form regulatory networks known as ceRNA. CircBCAR3 up-regulates transportin-1 (TNPO1) by combining with miR-27a-3p, which accelerates the tumorigenesis of esophageal cancer [11]. Several circRNAs have already been confirmed to be associated with lung cancer development [12,13]. The circUCP2/miR-149/UCP2 axis accelerates the progression of NSCLC [14]. New research has indicated that circular RNAs have an impact on the ferroptosis mechanism in malignant cells. The interaction between circRNA_101093 and fatty acid-binding protein 3 affects the sensitivity of treatment related to ferroptosis [15]. Regarding circ_0015278 (alternatively referred to as circ_100395), it is a newly discovered circRNA, and several researchers have found it inhibits to the development of tumors. A retrospective study of 206 patients with papillary thyroid carcinoma (PTC) found circ_0015278 was down-regulated in PTC, and the Receiver Operating Characteristic (ROC) curve analysis showed that the area under the curve was 0.903, and the 95% confidence interval was 0.874–0.932 [16]. Furthermore, low expression of circ_0015278 was associated with poor prognosis of PTC patients [16]. A bioinformatics analysis study combining multiple public data sets found that circ_0015278 was low expressed in NSCLC tissues [16]. In addition, many studies have shown that circ_0015278 could regulate the progress of NSCLC [17], ovarian cancer [18], and liver cancer [19] through ceRNA mechanism. However, the role of circ_0015278 in the ferroptosis mechanism of LUAD remains unexplored, underscoring the need for further investigation.

This study identified a novel circRNA (circ_0015278) associated with ferroptosis in LUAD. Our findings revealed decreased expression of circ_0015278 in LUAD, and its impact on cellular proliferation, migration, invasion, and ferroptosis in LUAD cells. Additionally, a xenotransplantation mouse model confirmed these findings, demonstrating that overexpression of circ_0015278 effectively inhibited LUAD growth. Furthermore, circ_0015278 downregulated the expression of SLC7A11 through the miR-1228/P53 signalling pathway, thereby enhancing ferroptosis in LUAD.

## Materials and Methods

### Data source

We obtained the microarray data from the Gene Expression Omnibus (GEO) database. Circ_0015278 expression was retrieved from GSE158695, GSE101586, GSE112214, and GSE146689 datasets. The expression profile of miR-1228 was obtained from the UALCAN (https://ualcan.path.uab.edu/) and GSE244311. To further verify the structural characteristics of circ_0015278, we referred to the Cancer-Specific CircRNA Database 2.0 available at http://geneyun.net/CSCD2/. In the upper part of the database page, we entered the parent gene, kelch like family member 20 (KLHL20) of circ_0015278 to search.

### Cell culture and transfection

The cell lines H1299 (catalog number: TCHu160), SPCA1 (catalog number: TCHu53), PC9 (catalog number: SCSP-5085), and A549 (catalog number: TCHu150), along with bronchial epithelial cells (HBEs), were acquired from the Shanghai-based Cell Bank of Chinese Academy of Sciences in China. These cells were cultured in RPMI-1640 medium (Solarbio, Beijing, China) supplemented with 1% penicillin/streptomycin (Solarbio, Beijing, China), and 10% FBS (Thermo Fisher Scientific, Waltham, MA, USA) at 37 degrees Celsius in 5% carbon dioxide atmosphere. Circ_0015278 overexpression plasmid were generated by GENESEED, a company based in Guangzhou, China using the pLC5-ciR carrier, with specific information available in the Fig. S1. Cells were transfected with circ_0015278 overexpression plasmid (pLC5/circ_0015278) or blank control plasmids (pLC5/vector). MiR-1228 mimics, miRNA controls, siP53, and siNC (control siRNA) were purchased from GenePharma in Shanghai, China. Transfection was performed using Lipofectamine 3000 reagent obtained from Thermo Fisher Scientific, Waltham, MA USA.

### RNA extraction and qRT-PCR

TRIzol (Thermo Fisher Scientific, MA, USA) was employed to extract total RNA and all qRT-PCR kits were obtained from GenePharma in Shanghai, China. Reverse transcription of RNAs was performed using the PrimeScript RT Master Mix following the manufacturer’s protocol. Subsequently, qRT-PCR was conducted on the Applied Biosystems™ 7500 real-time PCR system. GAPDH was used as the reference gene for standardizing circ_0015278 and P53 expressions, while U6 served as a reference for miR-1228 expression. Gene expression was calculated using the 2^−ΔΔCt^ method. Exact primer sequences are provided in Table S1.

### RNase R treatment and subcellular fractionation

An experiment was performed using LUAD cells to examinm the impact of 3 µ/µg RNase R (GENESEED, Guangzhou, China) on the expression of circ_0015278 and linear mRNA KLHL20. The expression levels of circ_0015278 and KLHL20 were assessed using qRT-PCR. The PARISTM Kit Protein and RNA Isolationg System (Pratt Biotechnology Co., Ltd, Hunan, China) was used following manufacturer’s guidelines to identify cellular localization of circ_0015278.

### FISH

According to the manufacturer’s protocol (GenePharma, Shanghai, China), circ_0015278 was hybridized with a Cy3-labeled probe, with DAPI utilized for nuclear staining. The probe sequence can be obtained in Table S2. The expression and localization of circ_0015278 in A549 and PC9 cells were observed using confocal laser scanning microscopy.

### CCK-8, colony formation, transwell, and wound-healing assay

Proliferation ability of LUAD cells was evaluated using a CCK-8 solution from obtained Beyotime, in Guangzhou, China. LUAD cells were seeded in 96-well plates (4 × 10^3^ cells per well). Cells were exposed to 10 μL of the CCK-8 reagent for 2 h at 24, 48, and 72-h intervals. Cell growth was assessed by measuring absorbance at 450 nm. Ferroptosis was induced in LUAD cells using Erastin (Beyotime, Guangzhou, China), with Dimethyl sulfoxide (DMSO) (SINOPHARM, Beijing, China) serving as the control.

For colony formation assays, LUAD cells (1 × 10^3^ cells/well) were transfected and seeded in 6-well plates, with the cell growth medium refreshed every three days. After two weeks, colonies were fixed with paraformaldehyde (Labcoms, Nanjing, China) and stained with crystal violet (Beyotime, Guangzhou, China).

To assess cell invasion, LUAD cells were subjected to serum deprivation in RPMI 1640 medium for 8 h and then seeded onto the superior cavity of pre-embedded matrigel (Corning, USA) (1 × 10^5^ cells) in a transwell system. After 18–24 h of culture, invading cells in the inferior cavity were fixed with paraformaldehyde, stained with crystal violet, photographed, and counted.

Scratch assays were performed by seeding LUAD cells (2 × 10^5^ cells per well) in 6-well plates and incubating them for 12 h. Scratches were made using a 100 μl pipet tip, and the cells were further incubated. Photographs were taken at 0 and 24 h to measure the distance migrated.

### Fe*^2+^*, ROS, GSH, and MDA assay

To determine Fe^2+^ levels, the Cell Ferrous Iron Colorimetric Assay Kit (Elabscience, Wuhan, China) was utilized, while ROS levels were assessed using the Reactive oxygen species Assay Kit (Biosharp, Hefei, China). The Fe^2+^ detection kit operates on the principle that ferrous ions in the sample react with the probe, generating a substance with a strong absorption peak at wavelength of 593 nm, with optical density linearly correlated with ferrous ion concentration within a certain range. Meanwhile, the ROS detection kit employs H2DCFDA probe, which permeates the cell membrane and is hydrolyzed by intracellular esterase to produce DCFH. Intracellular ROS then oxidise non-fluorescent DCFH to produce fluorescent DCF, enabling measurement of fluorescence intensity to determine intracellular ROS levels. GSH levels was were determined using the Total glutathione/Oxidized glutathione assay kit (Jiancheng, Nanjing, China), while MDA levels were assessed with Malondialdehyde (MDA) assay kit (Jiancheng, Nanjing, China). GSH serves as a crucial non-enzymatic antioxidant, involved in scavenging free radicals, facilitating iron absorption and maintaining erythrocyte membrane integrity. Conversely, MDA levels reflect the extent of lipid peroxidation in the body, with lipid peroxidation triggered by the attack of oxygen free radicals on polyunsaturated fatty acids (PUFAs) in the cell membrane, leading to the formation of lipid peroxides such as aldehyde groups (MDA) and ketone groups. Therefore, the amount of MDA measured often reflects the degree of lipid peroxidation in the body. All experiments adhered to the protocols provided by the reagent suppliers.

### Transmission electron microscopy

The researcher begins by directly scraping off the cells, followed by centrifuging the cells and culture solution in a pointed centrifuge tube at a speed of 2000 rpm for 5 min. After discarding, the supernatant 2.5% glutaraldehyde (dissolved in 0.1 M PBS, pH 7.4) (Phygene, Fuzhou, China) is added and the mixture is gently blown to ensure proper suspension. Subsequently, the cells are fixed overnight at 4°C. Next, they are soaked in a 1% osmium tetroxide solution (Phygene, Fuzhou, China), washed with a phosphate buffer, and dehydrated with ethanol before embedding in an epoxy resin. The samples are then cut into slices using an ultramicrotome (Leica, Wetzlar, Germany), stained with uranyl acetate and lead citrate, and finally observed using a transmission electron microscope (Hitachi, Tokyo, Japan).

### Luciferase reporter assay

To confirm the interaction, A549 and PC9 cells were transfected with reporter vectors containing Circ_0015278 or P53 3’UTR wild/mutant (GenePharma, Shanghai, China). MiR-1228 mimics or controls were introduced during the transfection process. Subsequently, a luciferase assay was performed using a luciferase kit (YEASEN, Shanghai, China) after 48 h of transfection to authenticate these interactions.

### Western blot analysis

The LUAD cell lines were treated with RIPA buffer (Santa Cruz, TX, USA) to facilitate the extraction of total cellular protein, followed by the utilization of the BCA Protein Assay Kit (Sangon, Shanghai, China) to quantitatively determine the protein content. Protein samples were then examined using a 12% SDS-PAGE. Subsequently, the proteins were transferred onto a PVDF membrane (YEASEN, Shanghai, China). The PVDF membrane was then subjected to an overnight incubation at 4°C with specific primary antibodies, including anti-GAPDH (1:2000, 60004-1-Ig, Proteintech), anti-p53 (1:1000, ab131442, Abcam, UK), and anti-SLC7A11 (1:1000, 26864-1-AP, Proteintech). The samples were further incubated with secondary antibody at room temperature.

### Xenograft tumor

10 female BALB/C mice (6–8 weeks old, weighing 19–22 g) (Jihui, Shanghai, China) utilized for the evaluation. Subcutaneous injection was administered in the left armpit of nude mice, ensuring the needle was inserted a slightly deeper under the skin, approximately 1 cm deep, to minimize the overflow of cell suspension post-injection. Tumor induction was conducted by subcutaneously injecting A549 cells (2 × 10^6^ cells) transfected with vector and circ_0015278 overexpression plasmid. Starting from the eleventh day, size of the cancer (calculated using the formula: width^2 × length × π/6) was measured at three-day intervals. On the 29th day, the tumor was surgically excised, and its weight was determined. All animal studies (including mouse euthanasia procedures) were carried out in compliance with regulations and guidelines for animal care of Soochow University Medical College, and according to AAALAC and IACUC guidelines (SCXK 2022-0009).

### Immunohistochemistry (IHC)

After dehydration, lung tissue sections underwent treatment with specific primary antibodies including anti-p53 (dilution ratio: 1:100, ab131442, Abcam, UK), and anti-SLC7A11 (1:200, 26864-1-AP, Proteintech) and were then incubated overnight at 4°C. Following this, HRP-coupled secondary antibodies IgG (Thermo Fisher Scientific, MA, USA) were added at a dilution ratio of 1:2000 and incubated at 37°C for 60 min. Positive staining was visualized using the DAB kit (UNYV, Shanghai, China). Finally, images of the stained samples were captured using a microscope.

### Statistical analysis

Statistical analysis was conducted using GraphPad Prism 8.0. The dissimilarities between two groups were evaluated using a two-tailed, unpaired Student’s *t*-test. For multiple comparisons, either a one-way or two-way ANOVA was performed. Statistical significance was determined based on *p*-values less than 0.05.

## Results

### Circ_0015278 was downregulated in LUAD tissues and cells

**Figure 1:**
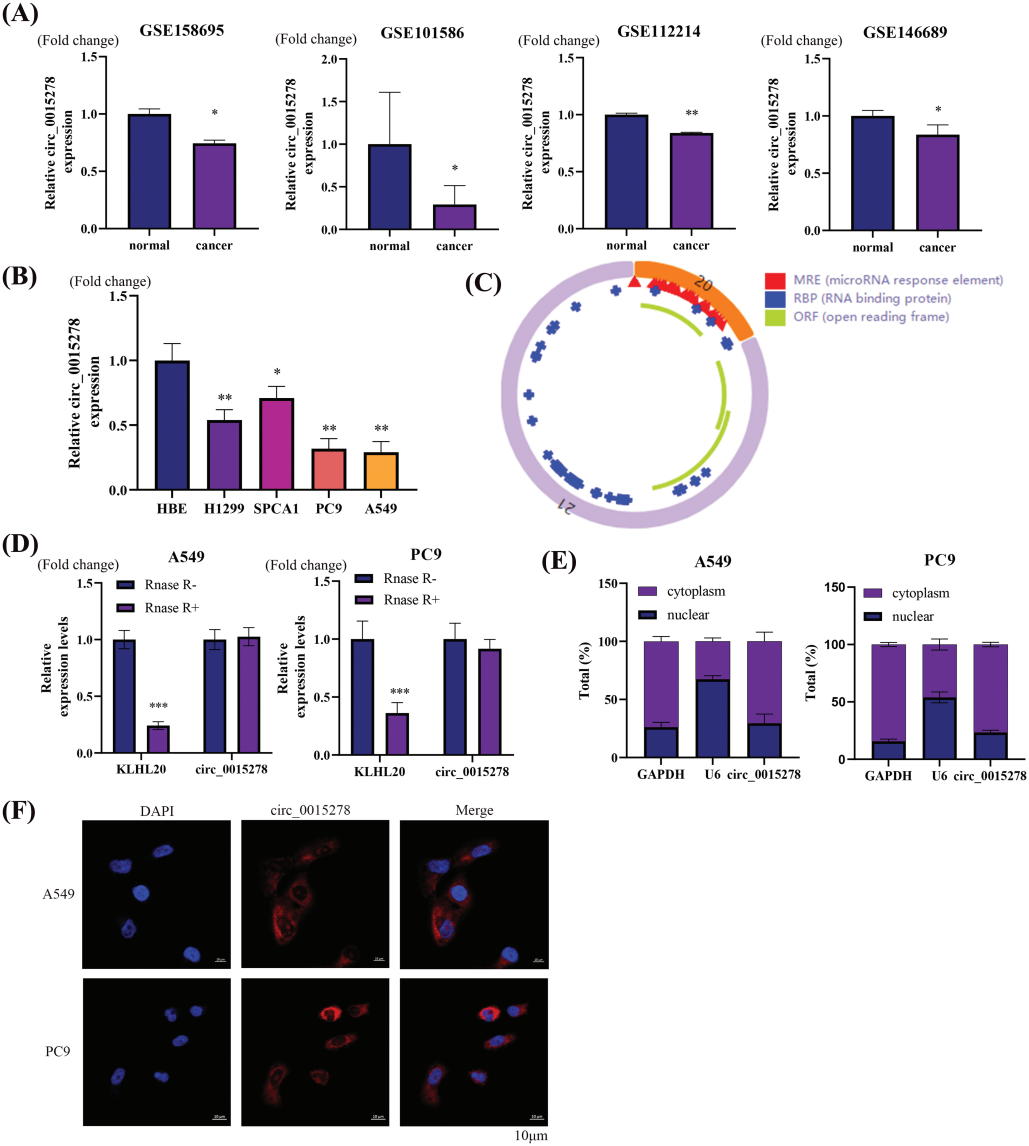
Circ_0015278 is downregulated in LUAD. (A) Circ_0015278 was downregulated in GSE158695, GSE101586, GSE112214, andGSE146689. (B) Circ_0015278 was downregulated in LUAD cell lines. (C) The schematic illustration of circ_0015278. (D) Circ_0015278 showed resistance against RNase R treatment. (E) The location of circ_0015278. (F) FISH showed the subcellular location of circ_0015278. *p < 0.05; **p < 0.01; ***p < 0.001.

Four distinct GEO datasets indicated a reduction in circ_0015278 expression in LUAD tissues (Fig. 1A). Notably, the expression profile of circ_0015278 in four LUAD cell lines closely mirrored that observed in lung cancer tissues (Fig. 1B). Our analysis confirmed circular structure of circ_0015278 and identified numerous microRNA response elements (Fig. 1C). Intriguingly, our study revealed that circ_0015278 exhibited increased resistance to RNase R compared to linear mRNA KLHL20 (Fig. 1D). To further investigate the localization of circ_0015278, we conducted nuclear-cytoplasmic separation experiments using LUAD cells, which confirmed its predominantly cytoplasmic presence (Fig. 1E). In addition, FISH analysis showed that circ_0015278 localized to the cytoplasm (Fig. 1F).

### Circ_0015278 inhibits cell proliferation, migration, and invasion

**Figure 2:**
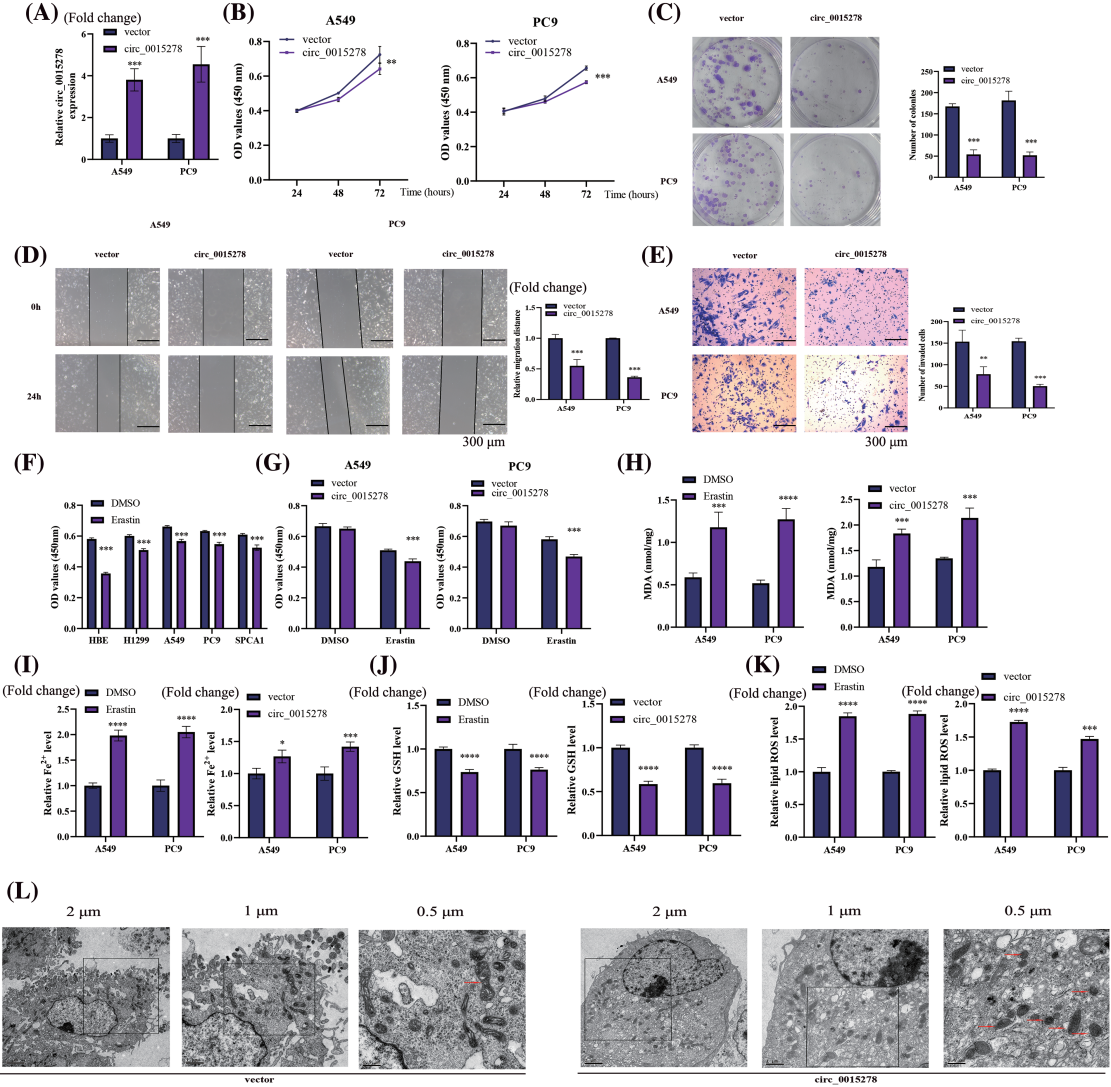
Overexpression of circ_0015278 affects malignant phenotype and ferroptosis of LUAD cells. (A) PCR analysis of LUAD cells transfected with circ_0015278 overexpression plasmid. CCK-8 (B), colony formation (C), wound healing (D) and transwell assays (E) were utilized to detect the malignant phenotype of LUAD cells. (F) The effect of erastin (10 µm, 24 h) on the viability of LUAD and HBE cells was evaluated by CCK-8. (G) The effect of overexpressing circ_0015278 on the viability of A549 and PC9 cells with erastin treatment (10 µm, 24 h) was measured by CCK-8. DMSO was employed as a control. MDA content (H), relative Fe^2+^ level (I), GSH content (J), ROS level (K), and was evaluated. (L) Mitochondrial morphology of A549 cells. *p < 0.05; **p < 0.01; ***p < 0.001; ****p < 0.0001.

To elucidate the biological role of circ_0015278 in LUAD, we transfected into A549 and PC9 cells with the circ_0015278 overexpression plasmid. Following transfection, a significant increase in circ_0015278 levels was observed (Fig. 2A). Subsequently, cell proliferation and colony formation assays revealed a detrimental effect of circ_0015278 overexpression on the viability and colony formation ability of A549 and PC9 cells (Fig. 2B,C). Furthermore, both wound healing and transwell experiments demonstrated that overexpression of circ_0015278 hindered the migratory and invasive capacities of LUAD cells (Fig. 2D,E).

### Circ_0015278 induces ferroptosis of LUAD cells

To induce the ferroptosis model, cells were treated with erastin (10 μM, 24 h). Our findings revealed that the decrease in the optical density (OD) value of HBE cells exceeded that of other LUAD cells, indicating a heightened resistance of LUAD cells to erastin (Fig. 2F). Following erastin treatment for 24 h, a significant reduction in cell viability was observed, with a further exacerbation of this effect noted following transfection of circ_0015278 overexpressed plasmid (Fig. 2G). To delve deeper into the impact of circ_0015278 on ferroptosis, we observed an increase in intracellular levels of MDA, Fe^2+^, and lipid ROS upon overexpression of circ_0015278, alongside a decrease in GSH levels, with erastin treatment serving as a positive control (Fig. 2H–K). Moreover, transmission electron microscopy revealed characteristic morphological changes associated with ferroptosis in A549 cells (Fig. 2L). Notably, in the group with overexpressed circ_0015278, mitochondria exhibited reduced size, increased membrane density with a decrease or absence of mitochondrial ridges.

### Circ_0015278 negatively regulates miR‑1228 in LUAD cells

**Figure 3:**
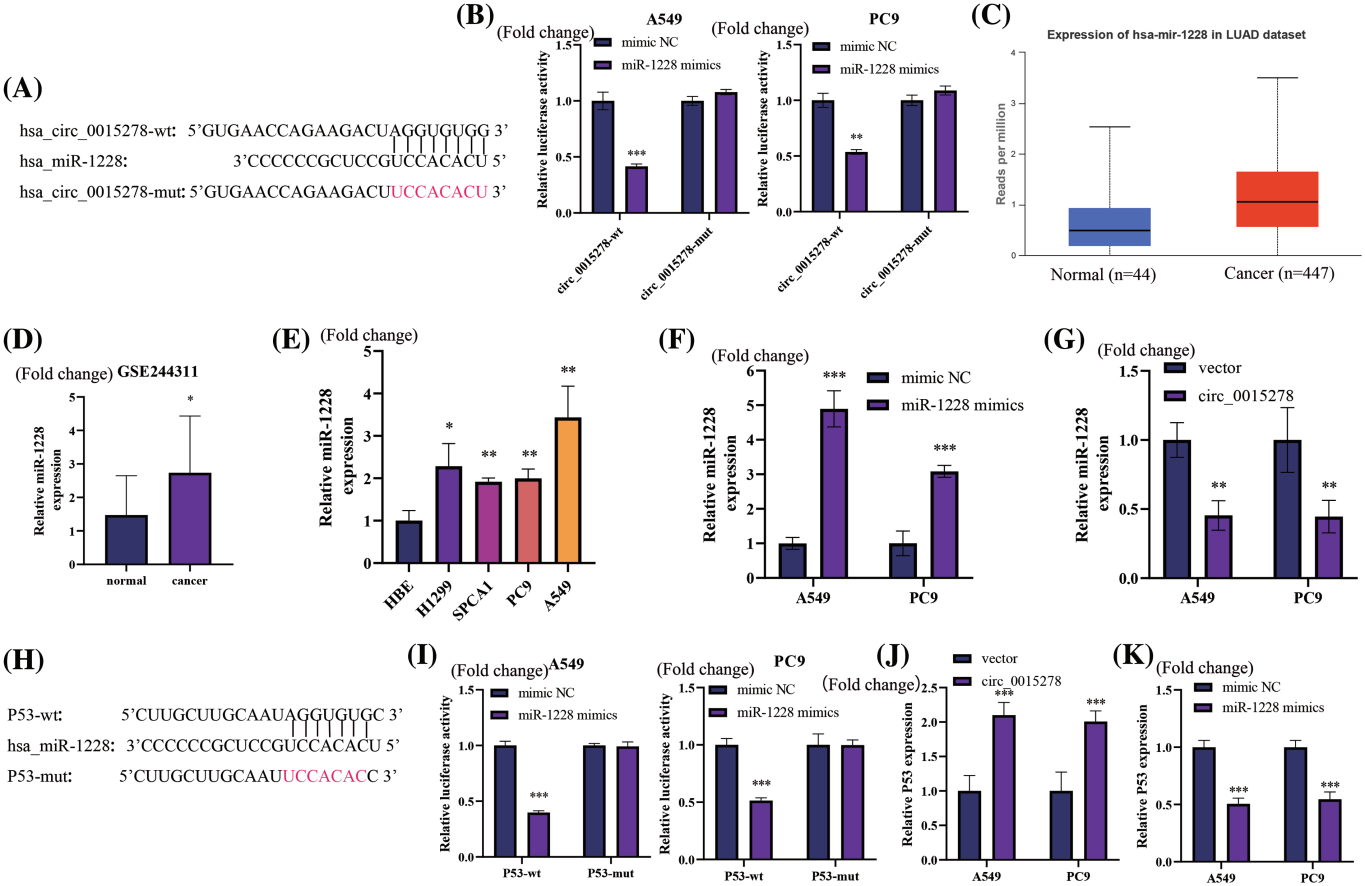
Circ_0015278 acts as the sponge of miR-1228 and P53 functions as miR-1228’s target in LUAD. (A) Binding sequences of miR-1228 on circ_0015278. (B) Luciferase reporter assays revealed miR-1228’s binding to circ_0015278. The expression of miR-1228 in UALCAN (C), GSE244311 (D), and LUAD cells (E). PCR tests in LUAD cells with miR-1228 upregulated (F) and circ_0015278 overexpression (G). (H) Binding sequences of miR-1228 and P53. (I) Luciferase reporter assays revealed miR-1228’s binding to P53. P53 expression in LUAD cells treated with circ_0015278 overexpression plasmid (J) and miR-1228 mimics (K). *p < 0.05; **p < 0.01; ***p < 0.001.

As per the CircInteractome (https://circinteractome.nia.nih.gov/index.html), a potential binding site of circ_0015278 with miR-1228 was identified (Fig. 3A). Upon overexpression of miR-1228, the fluorescence intensity of wild-type circ_0015228 significantly decreased, while the mutant circ_0015278 remained unaffected (Fig. 3B). Utilizing the UALCAN database and GSE244311, we observed high expression levels of miR-1228 in LUAD (Fig. 3C,D), which concurs with our findings from LUAD cell lines (Fig. 3E). The efficacy of miR-1228 mimics in promoting overexpression is depicted in Fig. 3F. Furthermore, the upregulation of circ_0015278 led to a reduction in miR-1228 levels in A549 and PC9 cells (Fig. 3G).

### P53 is a direct target of miR-1228 and positively regulated by circ_0015278

Based on data extracted from TargetScan database, a hypothesis was formulated suggesting that miR-1228 may have the ability to target P53, as depicted in Fig. 3H. The luciferase activity fluorescence intensity of the wild-type P53 significantly decreased upon overexpression of miR-1228, whereas the mutant P53 remained unaffected (Fig. 3I). Furthermore, the overexpression of circ_0015278 led to an increase in P53 expression within A549 and PC9 cells (Fig. 3J). Conversely, the overexpression of miR-1228 resulted in a significant decrease in the expression of P53, as demonstrated in Fig. 3K.

### The inhibitory effects of circ_0015278 were reversed by the introduction of miR-1228 mimic and siP53 in LUAD cells

**Figure 4:**
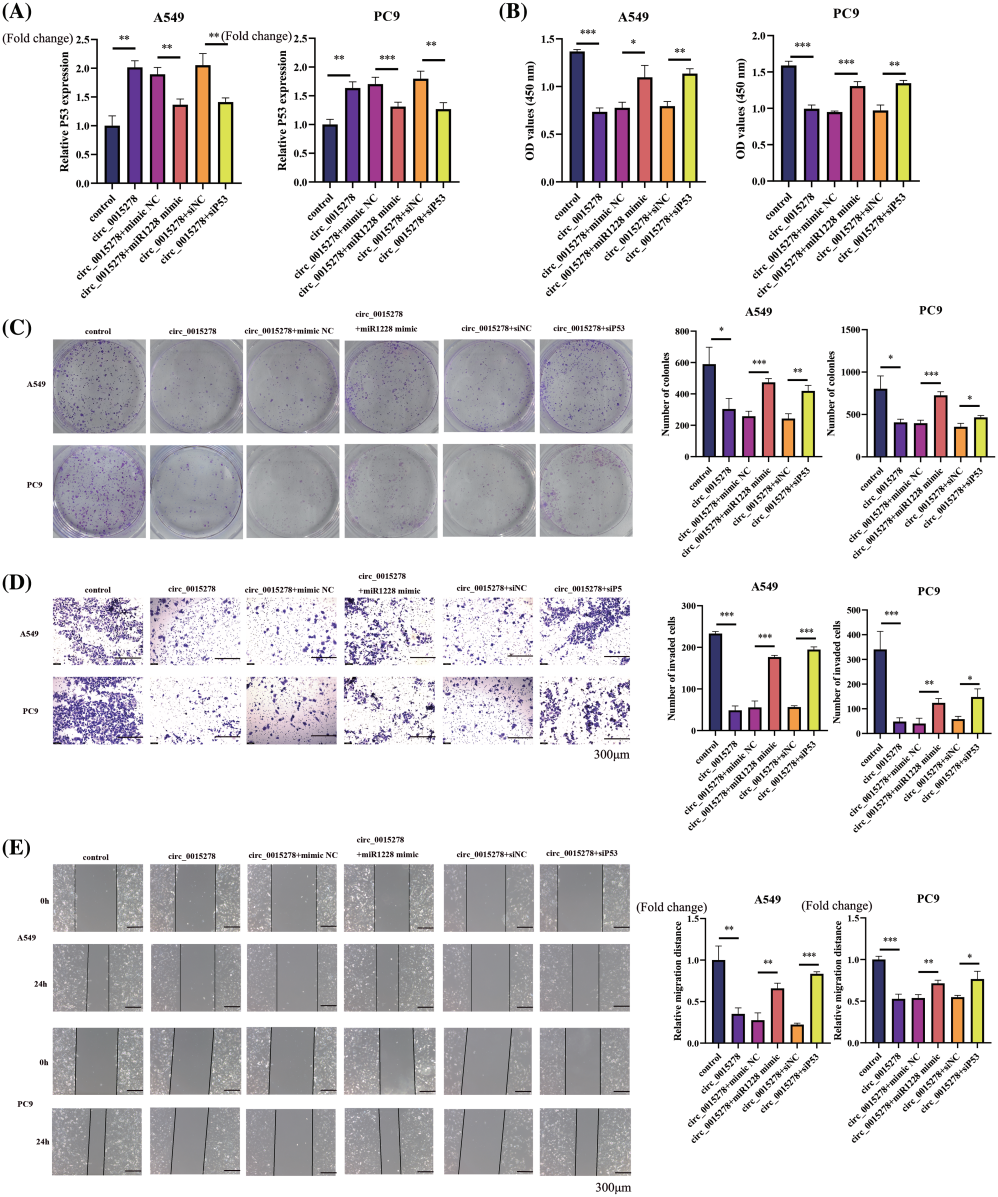
miR-1228 mimic and siP53 rescued the inhibitory effect mediated by circ_0015278. (A) mRNA levels of P53 in A549 and PC9 cells co-transfected with circ_0015278 and miR-1228 mimic or siP53. CCK-8 at 72 h after cell plating (B), colony formation (C), transwell assays (D) and wound healing (E) were utilized to detect malignant phenotype of LUAD cells. *p < 0.05; **p < 0.01; ***p < 0.001.

Subsequently, we explored the functional association between miR-1228, P53 and circ_0015278 in LUAD cells. Through qRT-PCR analysis, we observed that circ_0015278 upregulated P53 expression, while the introduction of miR-1228 mimic and siP53 decreased this effect (Fig. 4A). In line with previously reported findings, the overexpression of circ_0015278 significantly attenuated the malignant phenotype of A549 and PC9 cells. When miR-1228 mimic and siP53 were transfected, these effects partially reversed (Fig. 4B–E).

### Circ_0015278-induced ferroptosis can be reversed by miR-1228 mimic and siP53

**Figure 5:**
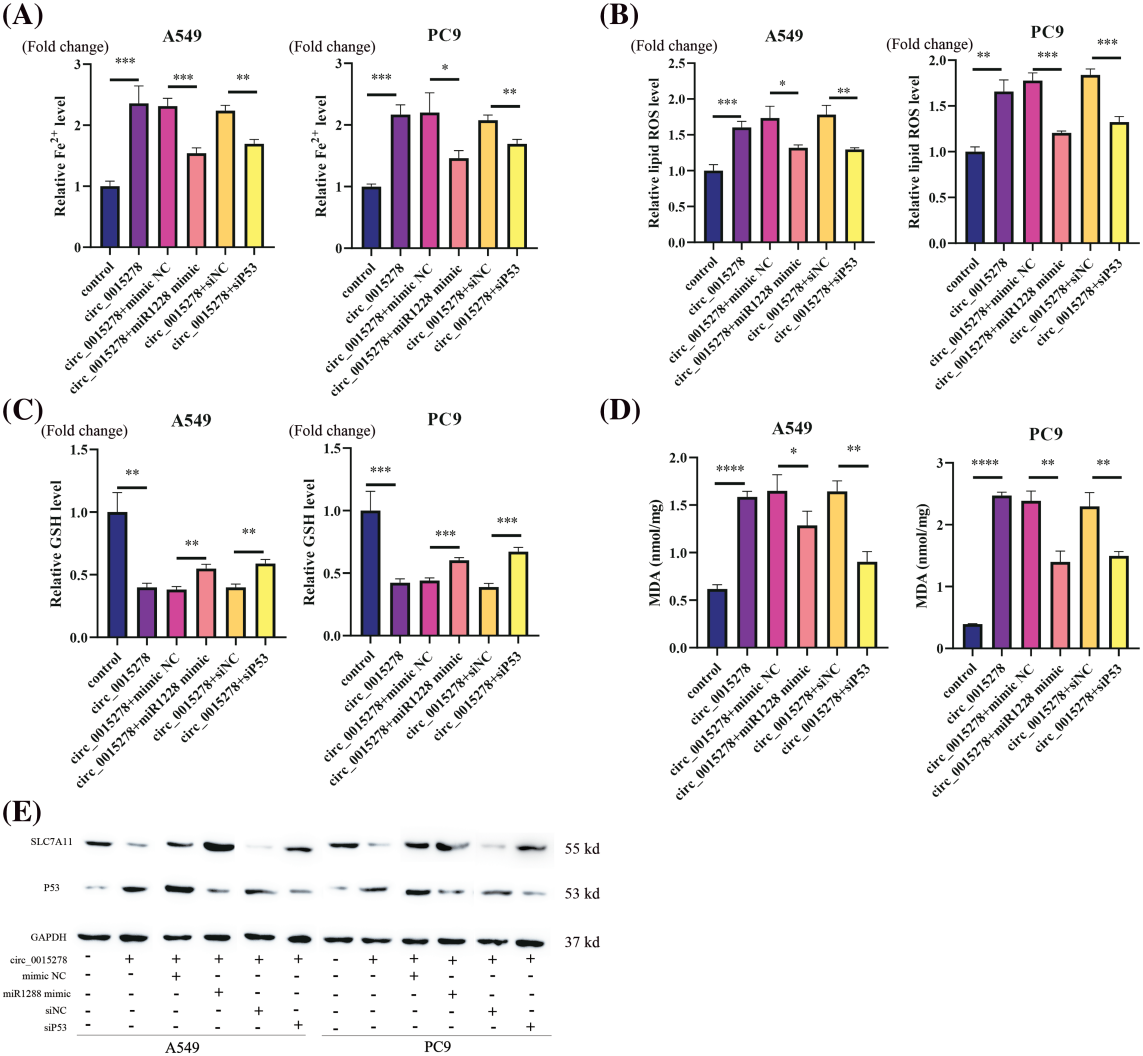
miR-1228 mimic and siP53 reversed circ_0015278 induced ferroptosis in LUAD cells. Relative Fe^2+^ level (A), ROS level (B), GSH content (C), and MDA content (D) was evaluated in LUAD cells. (E) Western blotting analysis of SLC7A11 and P53 levels. *p < 0.05; **p < 0.01; ***p < 0.001; ****p < 0.0001.

Subsequently, we investigated whether the introduction of miR-1228 mimic and siP53 could attenuate the ferroptosis induced by circ_0015278 in LUAD cells. Consistently, miR-1228 mimic and siP53 were observed to mitigate the effects of circ_0015278 on the relative content of MDA, levels of Fe^2+^, GSH, and ROS in A549 and PC9 cells (Figs. 5A–5D). According to previous research, P53 can suppress the expression of SLC7A11 to increase cell susceptibility to ferroptosis [19]. Through western blot analysis, we found that circ_0015278 upregulated P53 and downregulated SLC7A11. Additionally, the introduction of miR-1228 mimic and siP53 mitigated these effects (Fig. 5E). These findings collectively provide substantial evidence regarding the regulation of the circ_0015278/miR-1228/P53 axis.

### Overexpression of circ_0015278 inhibits the growth of LUAD in vivo

**Figure 6:**
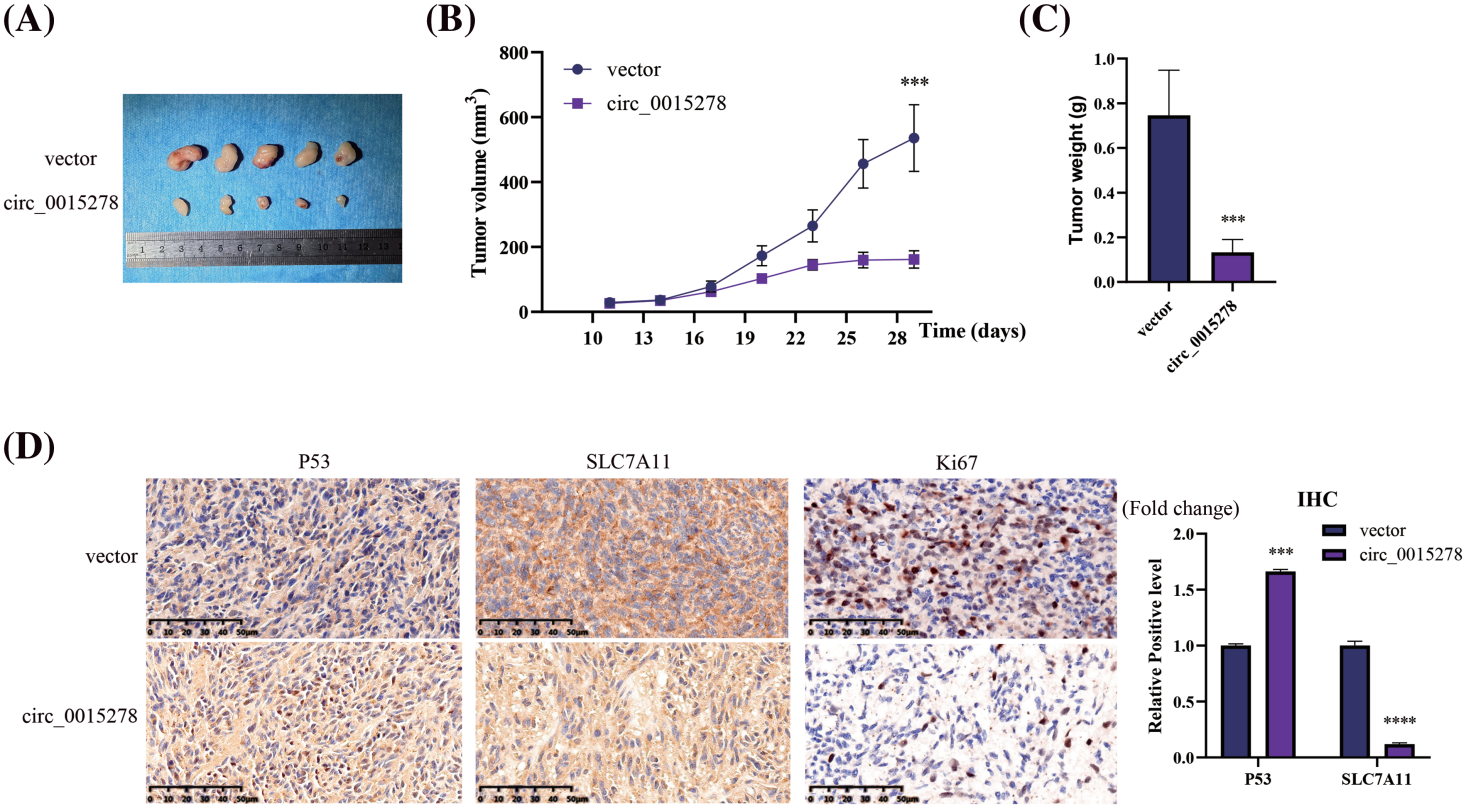
Circ_0015278 suppressed the LUAD growth *in vivo*. (A) Images of xenograft tumors. (B) The volume of tumor. (C) The weight of tumor. (D) IHC was utilized to assess the levels of SLC7A11 and P53 in the LUAD tissues. ***p < 0.001; ****p < 0.0001.

To assess the impact of circ_0015278 on tumor progression *in vivo*, we established a xenograft model in mice by injecting cells transfected with either vector or circ_0015278 overexpression plasmid. The results revealed that increased levels of circ_0015278 correlated with a reduction in both the size and weight of tumors in the mice (Fig. 6A–C). Immunohistochemistry (IHC) staining demonstrated that upregulation of circ_0015278 led to an increase in the expression of P53 while simultaneously suppressing the expression of SLC7A11 in the LUAD tissues of the mice (Fig. 6D).

## Discussion

Recent scientific research has highlighted the regulatory role of circRNAs in the malignant progression of various cancer types, including bladder cancer [20], colorectal cancer [21], hepatocellular carcinoma [22], breast cancer [23,24], and LUAD [25]. Our findings study unveiled a downregulation of circ_0015278 in LUAD cells, suggesting its potential tumour-suppressive properties and significant role in regulating malignant, phenotypes and ferroptosis in LUAD cells.

Circ_0015278 exhibits reduced expression levels in various tumour tissues and functions as a cancer cell suppressor. Existing evidence indicates that circ_0015278 inhibits breast cancer progression by targeting MAPK6 [26] and regulates the PI3K/AKT axis to impede gastric cancer progression [27]. Additionally, it controls epithelial-mesenchymal transition to suppress proliferation and metastasis in ovarian cancer [18] and hampers malignant transformation in NSCLC via the miR-141-3p/LatS2 axis [28] and the miR-1278/SOCS6 axis [17]. These findings collectively underscore circ_0015278’s role as a tumour suppressor gene, hindering tumour progression. Similarly, our study demonstrated that circ_0015278 inhibited malignant phenotypes in LUAD cells. Bioinformatics analysis suggests the potential involvement of circ_0015278 in the ferroptosis process of AML with FLT3-ITD mutation [29], although experimental validation was lacking. Our study presents the first evidence demonstrating the ability of circ_0015278 to induce ferroptosis in LUAD through a series of cell experiments.

Previous studies have confirmed the significant influence of miR1228 in various biological processes in cancer. Upregulation of miR-1228 promotes cell proliferation in gastric cancer [30]. By targeting the SCAI protein, miR-1228 facilitates the advancement of breast cancer [31]. Additionally, the promotion of hepatocellular carcinoma cell proliferation and metastasis is attributed to miR-1228 [32]. Moreover, the direct targeting of SCAI by exosome miR-1228 prompts the migration and invasion of osteosarcoma [33]. The above research results showed that miR1228 could promote the malignant process of tumor by regulating various target genes. Similarly, our findings indicated upregulation of miR-1228 in LUAD cells, regulating P53 gene expression and promoting cancer.

P53, a significant tumour suppressor gene, is among the most commonly mutated genes in human tumours [34]. Mutations in P53 are identified in over 50% of tumour cells, leading to aberrant P53 signaling pathway [35]. Concurrently, numerous circRNAs also regulate P53. CircWSB1 competes with the ubiquitination enzyme USP10, leading to P53 degradation and breast cancer progression [36]. CircNUDT21 promotes bladder cancer progression by modulating the miR-16-1-3p/MDM2/P53 axis [37]. CircKDM4C induces ferroptosis in acute myeloid leukemia through hsa-let-7b-5p/P53 axis [38]. The above research results have demonstrated the involvement of circRNAs in the regulation of the P53 gene. Similarly, circ_0015278 also increased P53 expression according to our study. Furthermore, it has been observed that miRNAs also play a role in regulating P53 expression. Down-regulation of P53 leads to the increase of miR-1228 expression. Conversely, miR-1228 negatively regulates P53 expression, forming a positive feedback loop between P53/miR-1228/P53 [32]. Similarly, we confirmed the binding target between miR-1228 and P53 through luciferase reporter gene experiments. Recent investigations indicate that P53 has a notable impact on ferroptosis, as it inhibits cystine intake by suppressing SLC7A11, a crucial element of the cystine/glutamate antiporter, making cells susceptible to ferroptosis [39]. We have observed that P53 enhanced ferroptosis in LUAD cells by modulating SLC7A11 expression.

Although many studies have found that circ_0015278 can affect the progress of lung cancer, its role in ferroptosis has not been elaborated in detail. Therefore, our research focused on the role of circ_0015278 in the ferroptosis process of LUAD. In conclusion, our findings reveal a negative correlation between circ_0015278 and LUAD. Additionally, circ_0015278 upregulation hindered progression LUAD and induced ferroptosis by modulating miR-1228 and P53 activities. In addition, the structural diagram of circ_0015278 predicted that it has an open reading frame and may be able to encode peptides. We will explore this possible mechanism in depth in future studies. Exploring the regulation of the circ_0015278/miR-1228/P53 axis is crucial for advancing potential therapeutic strategies for LUAD.

## Supplementary Materials

Figure S1 Schematic diagram of the structure of plasmid used.

## Data Availability

The data that back up the findings of this article are provided in the article. For more information, please contact the authors who are corresponding.

## Supplementary Materials

The supplementary material is available online at https://doi.org/10.32604/or.2024.050835.
