# Supplementary figures and images for: CircRNA circ_0015278 induces ferroptosis in lung adenocarcinoma through the miR-1228/P53 axis
Source: Oncol Res. 2025 Jan 16;33(2):465–75. doi: 10.32604/or.2024.050835 (PMC11753987; doi:10.32604/or.2024.050835)

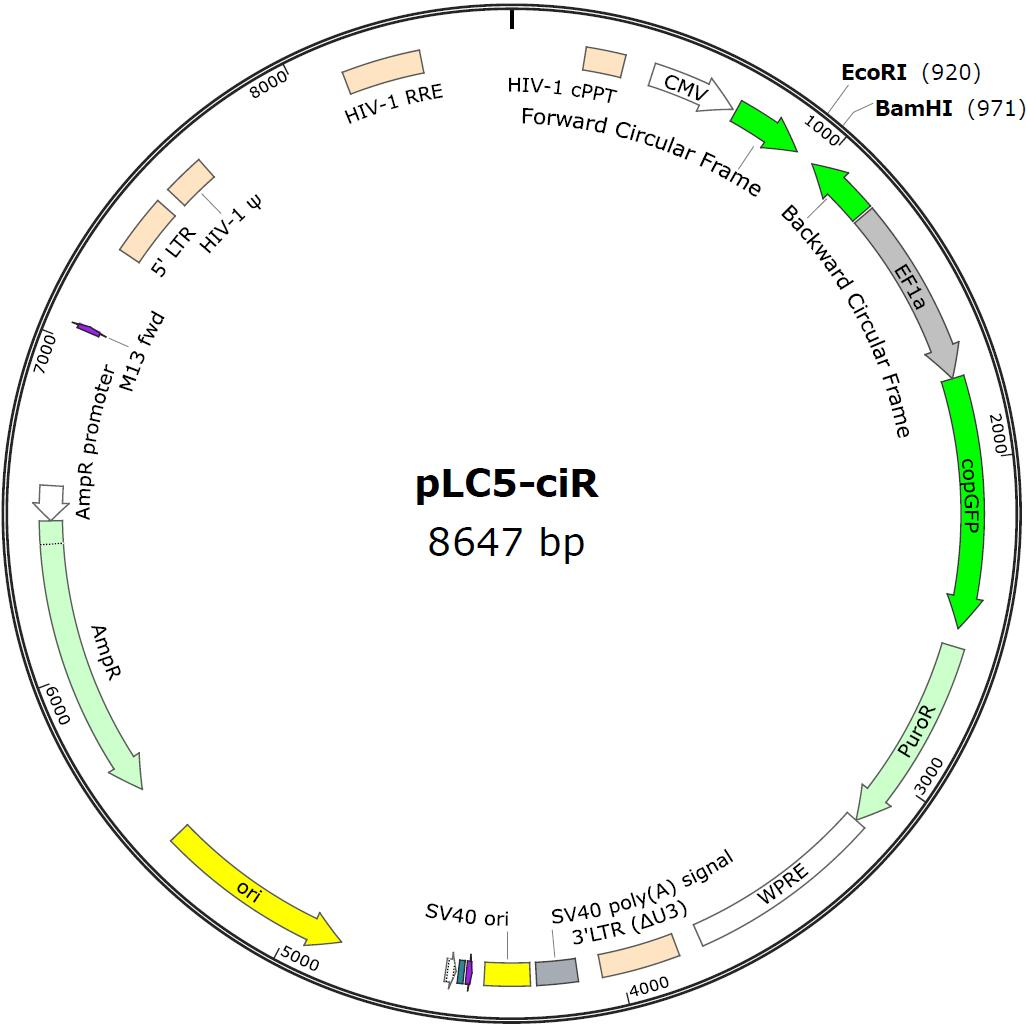

Supplement: Figure S1 [file OncolRes-33-50835-s001.tif]
