# Supplementary material for: CircRNA circ_0015278 induces ferroptosis in lung adenocarcinoma through the miR-1228/P53 axis
Source: Oncol Res. 2025 Jan 16;33(2):465–75. doi: 10.32604/or.2024.050835 (PMC11753987; doi:10.32604/or.2024.050835)
Supplement: Supplementary file 2 [file OncolRes-33-50835-s002.docx]

**Supplementary Table 1.** Exact primer sequences for qRT-PCR.

| **Gene** | **Primer sequences(5'-3')** |
| --- | --- |
| circ_0015278 | Forward: 5'-ATGACATCACGCCGTAGTGG-3' Reverse: 5'-CCCAACTCCGCATCTCCTTT-3' |
| miR-1228 | Forward: 5'-TCTATCTCTGTCACACCTGCCTCG-3' Reverse: 5'-GTGCAGGGTCCGAGGT-3' |
| P53 | Forward: 5'-GTACCACCATCCACTACAACTACAT-3' Reverse: 5'-AAACACGCACCTCAAAGCTG-3' |
| GAPDH | Forward: 5'-CATGAGAAGTATGACAACAGCCT-3' Reverse: 5'-AGTCCTTCCACGATACCAAAGT-3' |
| U6 | Forward: 5'-CTCGCTTCGGCAGCACA-3' Reverse: 5'-AACGCTTCACGAATTTGCGT-3' |
