# Supplementary material for: CircRNA circ_0015278 induces ferroptosis in lung adenocarcinoma through the miR-1228/P53 axis
Source: Oncol Res. 2025 Jan 16;33(2):465–75. doi: 10.32604/or.2024.050835 (PMC11753987; doi:10.32604/or.2024.050835)
Supplement: Supplementary file 3 [file OncolRes-33-50835-s003.docx]

**Supplementary Table 2.** Sequence information of probes used in FISH experiments.

| **Probe** | **Sequence** |
| --- | --- |
| circ_0015278 probe | 5' TTAT+TGTGCATCT+TGCAGAAACTCT 3' |
| NC probe | 5' TGCTTTGCACGGTAACGCCTGTTTT 3' |
